# The Butchers and Tuberculous Meat

**Published:** 1900-08-04

**Authors:** 


### Editor's Letter-Box.

[Our Correspondents are reminded that prolixity is a great bar to publication, and that brevity of style and conciseness of statement greatly facilitate early insertion.]

### THE BUTCHERS AND TUBERCULOUS MEAT.

W. A. Bond, M.A., M.D., Medical Officer of Health for the Holborn District, writes: I am much obliged to you for kindly sending me a copy of your last issue containing an annotation on the above subject. The carcase in question was fat and in good condition, and there was no evidence of tuberculosis in any part of it. In the Holborn District we act in accordance with the recommendations of the Royal Commission on Tuberculosis, and I therefore advised Dr. Sykes not to seize the carcase. P.S.—Kindly insert this in your next issue as my name was mentioned.

*** In commenting on this case we stated, as Dr. Bond now repeats, that "the carcase appeared well fed and healthy to the naked eye." The whole interest of the question, indeed, hinged upon that point, namely, whether an apparently healthy carcase of a beast whose viscera were considerably affected in both cavities was to be passed and condemned. If Dr. Bond will refer to our article he will observe that we did not comment on the practice in the Holborn District, but merely quoted the reason given by Dr. Sykes for not seizing the carcase. In regard to the recommendations of the Royal Commission on Tuberculosis, it may be worth while in this connection to point out that their recommendation was that the carcase if otherwise healthy (which appeared to be the case in this instance) shall not be condemned, but every part of it containing tuberculous lesions shall be seized (*a*) when the lesions are confined to the lungs (which was not the case here); (*b*) when the lesions are confined to the liver (which was not the case here); (*c*) when the lesions are confined to the pharyngeal lymphatic glands (which was not the case here); and (*d*) when the lesions are confined to any combination of the foregoing but are collectively small in extent, which certainly was not the case in this instance, seeing that both lungs contained "numerous large and small tubercular nodules" and the liver "numerous tubercular nodules, both large and small," and that in addition to there being "pronounced tuberculosis" in the pharyngeal lymphatic glands, the same condition was also found in the mesenteric glands. We go by the published account, but clearly such lesions as are there mentioned cannot be called "collectively small in extent," and thus a carcase containing the lesions described by Dr. Sykes does not come into any one of the four categories as to which the Commission recommends that the carcase shall not be condemned, although, on the other hand, it may not fall into any one of those in regard to which it is definitely stated that the entire carcase and all the organs shall be seized. The very point is that in such a case as this, in which admittedly there was a difference of opinion, the trader rather than the consumer got the benefit of the doubt, and that although the medical officer of health himself said that he "came to the conclusion that this animal was suffering from general tuberculosis,"* he nevertheless, after consultation, passed the meat, giving as his grounds for so doing that the "evidence as to local trade practice would in all probability have led a magistrate to decide against condemnation, and consequently very costly legal proceedings might have ensued, either upon appeal by the vestry or upon action for damages by the owner."—Ed. *T.H.*

* Report in "Public Health," July, 1900.

